# Development and Validation of an ELISA for the Detection of Bluetongue Virus Serotype 4-Specific Antibodies

**DOI:** 10.3390/v13091741

**Published:** 2021-08-31

**Authors:** Emmanuel Bréard, Mathilde Turpaud, Georges Beaud, Lydie Postic, Aurore Fablet, Martin Beer, Corinne Sailleau, Grégory Caignard, Cyril Viarouge, Bernd Hoffmann, Damien Vitour, Stéphan Zientara

**Affiliations:** 1 UMR 1161 Virologie, Laboratory for Animal Health, INRAE, Department of Animal Health, Ecole Nationale Vétérinaire d’Alfort, ANSES, Université Paris-Est, 94700 Maisons-Alfort, France; mathilde.turpaud@anses.fr (M.T.); Georges.BEAUD@vet-alfort.fr (G.B.); lydie.postic@anses.fr (L.P.); aurore.fablet@vet-alfort.fr (A.F.); corinne.sailleau@anses.fr (C.S.); gregory.caignard@vet-alfort.fr (G.C.); Cyril.VIAROUGE@anses.fr (C.V.); damien.vitour@vet-alfort.fr (D.V.); stephan.zientara@anses.fr (S.Z.); 2 Institute of Diagnostic Virology, Friedrich-Loeffler-Institut, 17493 Greifswald-Insel Riems, Germany; martin.beer@fli.de (M.B.); bernd.hoffmann@fli.de (B.H.)

**Keywords:** bluetongue, serotype-specific ELISA, serotype 4

## Abstract

In this article, we describe the development and evaluation of a double antigen sandwich enzyme-linked immunosorbent assay (ELISA) able to detect serotype 4-specific antibodies from BTV-4 infected or vaccinated animals using a recombinant BTV-4 VP2 protein. The coding sequence of VP2 was inserted into a pVote plasmid by recombination in the Gateway^®^ cloning system. Vaccinia virus (VacV) was used as a vector for the expression of the recombinant VP2. After production in BSR cells, recombinant VP2 was purified by immunoprecipitation using a FLAG tag and then used both as the coated ELISA antigen and as the HRP-tagged conjugate. The performance of the ELISA was evaluated with 1186 samples collected from BTV negative, infected or vaccinated animals. The specificity and sensitivity of the BTV-4 ELISA were above the expected standards for the detection of anti-BTV-4 VP2 antibodies in animals reared in Europe or in the Mediterranean basin. Cross-reactions were observed with reference sera for serotypes 10 and 20, and to a lesser extent with serotypes 12, 17 and 24, due to their genetic proximity to serotype 4. Nevertheless, these serotypes have never been detected in Europe and the Mediterranean area. This ELISA, which requires only the production of a recombinant protein, can be used to detect BTV serotype 4-specific antibodies and is therefore an attractive alternative diagnostic method to serum neutralization.

## 1. Introduction

Bluetongue (BT) is a non-contagious, OIE-listed disease that infects domestic and wild ruminants [1]. The disease is caused by viruses (BTV) transmitted from one animal to another by biting midges (*Culicoides* spp.). Some BTV strains can also be transmitted vertically or orally [2]. The disease is most commonly expressed in sheep, and occasionally in goats and certain deer species. During epizootics in northern Europe, BTV serotype 8 (BTV-8) also causes clinical signs in cattle [3]. Since the beginning of the 20th century, 9 of the 24 conventional serotypes (1, 2, 3, 4, 6, 8, 9, 11 and 16) have been detected in Europe [4,5,6,7], as well as serotypes 25, 27 and 32 [8,9]. These three BTV strains belong to the class of BTV serotypes found in asymptomatic small ruminants [4,8]. Therefore, in the European Union, animals infected with these non-virulent BTV strains are not reportable and do not lead to any restrictions on animal trade. Since the 2000s, inactivated vaccines have been available in Europe, against serotypes 1, 2, 4, 8 and 16, and are mainly used for trade [4]. To date, 35 confirmed BTV serotypes have been identified worldwide [8].

Five serotypes have been circulating in France for the past 20 years. As a result, several serotypes can be present at the same time and in the same place, as observed in France in 2008–2010 (BTV-1 and 8) and since 2018, with BTV-8 and 4 considered endemic [4].

BTV has a genome of 10 double-stranded RNA segments encoding 7 structural (viral protein 1 (VP1) to VP7) and 5 non-structural proteins (NS1-NS4 and NS3a) [1,10]. BTV serotypes are recognized on the basis of specific interactions between neutralizing antibodies (NAs) and VP2 of the outer capsid [11,12], which induce NA production after vaccination or infection [11]. VP7 is the major component of the inner capsid and is the BTV group-specific protein used as the antigen in serologic diagnostic tools, including enzyme-linked immunosorbent assays (ELISAs) [12]. Determination of BTV serotype by serological analyses can be performed only by seroneutralization testing (SNT) that require use of cell culture, live virus and several days of incubation. Moreover, previous studies have described serological relationships between serotypes circulating in Europe (1, 2, 4, 8 and 9) and partial cross-neutralization between these serotypes [13,14]. When at least two of these serotypes are present in the same area, as is currently the case in France with BTV-4 and 8, antibody typing by SNT may be difficult to interpret.

In order to determine the presence of serotype-specific antibodies, it would be useful to perform a simple technique such as ELISA with the same performance as SNT, in order to gain in handling time and especially in response time. This would make it possible to obtain a diagnosis in a few hours rather than in 5 to 6 days, due to the incubation time associated with SNT. A previous study showed that a sensitive and specific microsphere immunoassay for simultaneous group and serotype detection of BTV antibodies was as specific as SNT [15]. However, the technology used is difficult to implement compared to an ELISA test, in which a simple optical density reader is required. Therefore, we decided to develop an ELISA for the detection of the serotype-specific antibodies using a recombinant BTV VP2.

This article describes the development, evaluation and validation of a double-antigen sandwich ELISA able to characterize BTV-4 infected or vaccinated animals. This format provides an advantage because it detects total rather than class-specific antibodies. Vaccinia virus (VacV), an Orthopoxvirus in the Poxvirus family, was selected as the vector for VP2 recombinant protein production [16,17].

## 2. Materials and Methods

### 2.1. Sera Tested

The species, origin and sampling year of the 1186 sera tested in this study are presented in Table 1. Some of the sheep, goats and cattle sampled in the field in mainland France could be naturally infected with BTV-8 and/or BTV-4 when sampled after 2017. The ruminants sampled at the zoo of Beauval, France, were vaccinated with both inactivated BTV-4 and BTV-8 vaccines. In addition, 180 BTV-4 and BTV-8 goats vaccinated and sampled 3 weeks after the 2nd injection of a bivalent vaccine were analyzed in this study. The samples from cattle, goats and sheep used in the different experiments were naïve, vaccinated and/or challenged during experiments involving BTV vaccinations and homologous or heterologous challenges (Table 1).

The specificity of the ELISA was investigated by testing 625 serum samples. Specificity was first assessed in the presence of 319 sera from BTV-naïve ruminants collected in mainland France prior to the 2006 BTV-8 epizootic and/or tested negative by c-ELISA. Cross-reactivity with epizootic hemorrhagic disease virus (EHDV) was assessed with reference sera representing each EHDV serotype. Specificity was also tested on 306 sera from BTV-1, 2, 8, 9 or 16 infected and/or vaccinated animals (Table 1). Reference sera (57 sera representing 29 of the 35 BTV characterized serotypes [8]) were also analyzed with the BTV-4 ELISA.

Their status was defined by cELISA (when only serotype 8 was present in France, before 2017) and/or BTV-4 and 8 SNT and/or PCR or by the BTV epidemiological situation in the areas where they lived.

### 2.2. Cells, Plasmids and Viruses

BSR cells were used for infections and viruses were titrated by limiting dilution. The virus MVA-T7green (expressing IPTG-inducible T7 RNA polymerase) derives from MVA-EM24 [23] by insertion of the gene encoding the eGFP under the control of a synthetic early/late promoter and also by insertion of a gyrB-PKR cassette [24] into the MVA hemagglutinin locus as well as a gene encoding the mCherry protein under the control of the same synthetic early/late promoter (R. Drillien, personal communication).

The amplified gene encoding BTV-4 VP2 (Corsican strain isolated in 2016) was cloned using Gateway^®^ cloning technology (Invitrogen, Waltham, MA, USA). In short, 500 ng of pDONR207 vector was mixed with 2 µL of PCR product and 2 µL of BP Clonase™ in a final volume of 10 µL. The pDONR207 construct was amplified after transformation of E. coli DH5α (NEB) and extracted from bacteria using a NucleoSpin Plasmid kit (Macherey Nagel, Duren, Germany) following the manufacturer’s instructions [25].

The VP2 cDNA cloned into pDONR207 was transferred into the vaccinia virus Gateway destination vector pVote–FGW plasmid [15] (N-terminal tag FLAG) with LR Clonase™ and transformation of E. coli DH5α. Plasmids from 4 colonies were purified and each plasmid was tested for VP2-Flag expression. To this end, BSR cells were infected at a multiplicity of infection (MOI) of 3 plaque forming units (PFU) with MVA-T7green and transfected one hour later with plasmid according to the manufacturer’s instructions (JetPrime, Thermofisher, Waltham, MA, USA) and incubated at 37 °C overnight. Whole cell extracts were tested by Western blot with an anti-FLAG (Sigma, St. Louis, MO, USA) for production of VP2-Flag. Transfection of all four plasmids led to VP2-Flag expression and one of them was chosen to construct MVA-T7green-VP2. To this end, BSR cells were infected with MVA-T7green at MOI of 0.05 PFU, transfected with pVote2–FGW-VP2 and incubated at 37 °C for 2 to 3 days. MVA-T7green-VP2 recombinants were then obtained by gpt selection [26] followed by two successive rounds of coumermycin counter-selection [24].

### 2.3. VP2 Expression and Purification

T75 flasks with confluent BSR monolayers were inoculated with an MOI ranging from 1 to 10. After absorption for 1 h, 15 mL of culture medium, supplemented with 10mM IPTG, were added. After 24 h at 37 °C, the culture medium was removed, the cell layer was washed twice with PBS, and 1 mL of PBS containing anti-proteases (EDTA-free Protease Inhibitor Cocktail, Roche, Basel, Switzerland) was put on each T75 monolayer. The infected BSR cells were scraped off and all were put in a 2 mL microtube and frozen at −20 °C.

The microtube was thawed, vortexed and centrifuged at 10,000× *g* for 10 min at 4 °C. The supernatant was collected and added to 20 µL of anti-Flag M2-Agarose Affinity Gel from a Flag Immunoprecipitation kit (Sigma) following the manufacturer’s recommendations. The recombinant protein captured by the resin was then eluted, in the presence of a 3-fold higher concentration than recommended, by the 3X FLAG peptide. A fraction of the elution was analyzed by SDS-PAGE to confirm the presence of the recombinant protein (Western blot (WB) using anti-Flag) and to estimate its purity (Coomassie staining).

The purified VP2 was then used as an antigen (in this case 20% glycerol was added to the purification product for storage at −20 °C) or was labelled with peroxidase.

### 2.4. VP2 Labelling with HRP

Purified VP2 protein (600 µL), derived from the induction of 3 T75 flasks infected with recombinant MVA, was placed on the membrane of a centrifugal filter device (Amicon^®^ Ultra 30K (Millipore, Burlington, MA, USA)) to remove the 3X FLAG peptide. Then, 100 µL of 100 mM NaCl was added to the purified VP2, before being centrifuged at 7000× *g* until the volume was less than 100 µL. One milliliter of anti-protease PBS with 10 mM NaCl was added to the membrane, and the column was centrifuged at 7000× *g* until a volume of 100 µL was obtained, which was then used to label the VP2 with an HRP Conjugation kit (Abcam, Cambridge, UK), following the manufacturer’s instructions.

### 2.5. VP2 BTV-4 ELISA

The optimal dilution of VP2 antigen and VP2-HRP was determined by performing a checkerboard titration of antigen and VP2 HRP in the presence of BTV-4 positive (sera sampled at D17 or D63 from cattle experimentally infected with BTV-4) or negative ruminant sera. Based on these assays, antigen was diluted 1:200 in PBS when VP2-HRP (1:2500) and sera (1:10) were diluted in ELISA buffer “No 14” (Innovative Diagnostics, Grabels, France) diluted 1:4 in PBS.

Microtiter plates (Maxisorb) were coated (40 μL/well) and incubated overnight at 4 °C. Following one wash with PBS, 50 μL/well of diluted serum samples was added and incubated for 3 h at 4 °C. Plates were washed 3 times with PBS containing 0.1% Tween-20 and 40 μL/well of VP2-HRP was added and incubated for 45 min at room temperature (RT). After 4 washes, 75 μL of TMB reagent (Sigma) was added to each well. Plates were incubated at RT for 10 min and the reaction stopped with 75 μL of H_2_SO_4_ (0.5 M). The optical density (OD) was measured at 450 nm. ELISA results were expressed as raw optical density. The results presented are from assays where each serum was analyzed only once, as in an ELISA diagnostic setting.

## 3. Results

### 3.1. VP2 Expression and Purification

**Figure 1:**
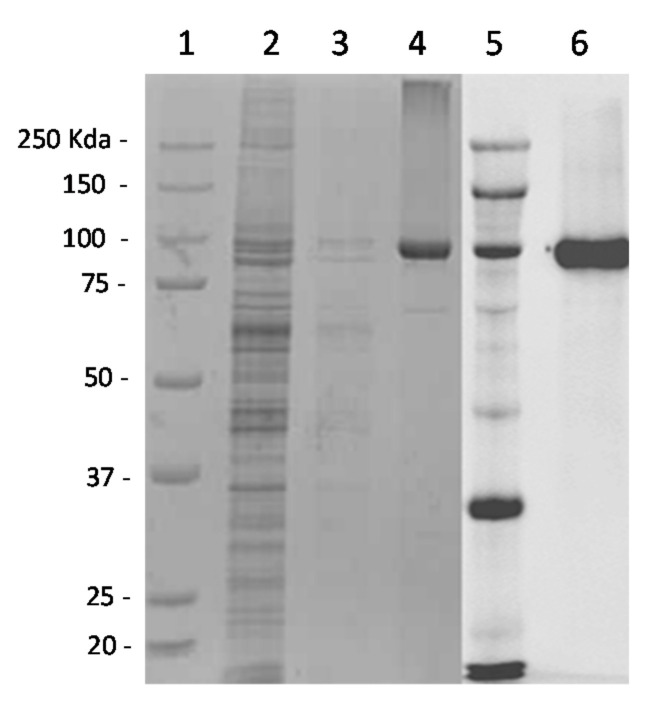
SDS acrylamide gel 10% (tracks 1 to 4) and Western blotting (tracks 5 and 6). Track 1 and 5: Marker Precision Plus protein westernC standard (Biorad, Hercules, CA, USA); track 2: PBS supernatant fraction; track 3: wash fraction; track 4 et 6: Fraction eluted with the Flag peptide.

After induction in the presence of IPTG, the infected cells contained the recombinant protein and as described above, a fraction of VP2-Flag was found in a soluble state in supernatants of infected cells that had been suspended in PBS, freeze–thawed and centrifuged to remove cell debris (Figure 1, column 2). After purification using anti-Flag agarose matrix, the eluted fraction contained the VP2-Flag as shown by Coomassie blue staining of SDS-PAGE gel and Western blotting using a mouse anti-FLAG antibody (Sigma) (Figure 1, columns 4 and 6, respectively).

### 3.2. ELISA Results on Naïve or BTV-1, 2, 8, 9 or 16 Infected or Vaccinated Animals

**Figure 2:**
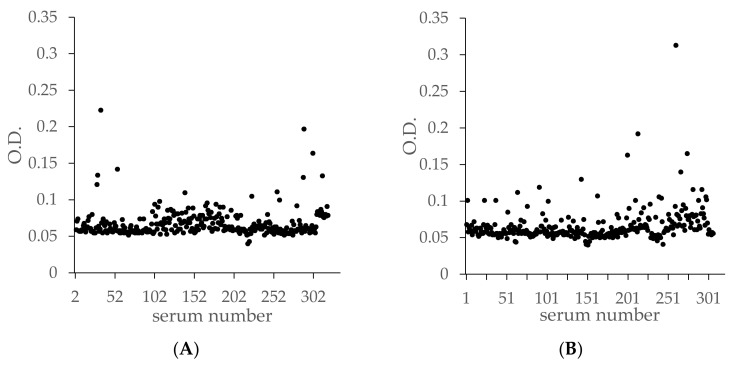
OD ELISA results from naïve (**A**) or BTV-1, 2, 8, 9 and 16 infected or vaccinated sera (**B**).

A vast majority of the 319 BTV-naïve ruminant sera had an OD of less than 0.1. Only three sera (1%) had an OD between 0.15 and 0.25 (Figure 2A). All seven sera representing the seven serotypes of EHD were negative. Similar results were obtained in the presence of 306 sera from animals vaccinated or infected with serotypes 1, 2, 8, 9 and 16. Here, four sera had an OD > 0.15, including one (representing serotype 1) with an OD greater than 0.25 (0.302).

### 3.3. ELISA Results on Experimental or Field BTV-4 Infected Ruminants

**Figure 3:**
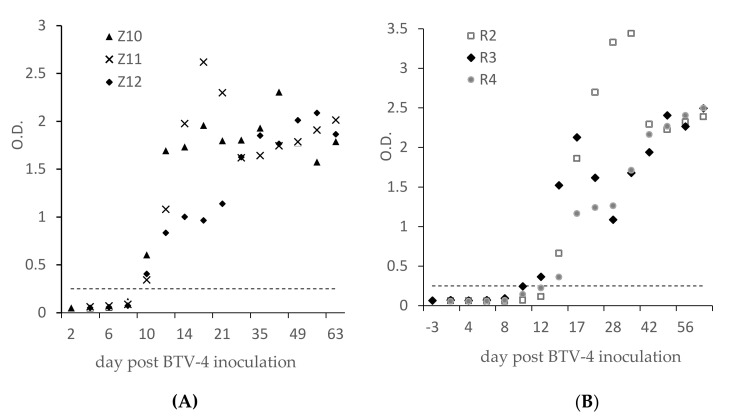
OD ELISA results from experimentally BTV-4 infected animals. Three goats (Z) (**A**) and three cattle (R) (**B**) were infected with the Bulgarian BTV-4 strain [19] at D0. The horizontal line represents the ELISA cut-off (OD = 0.25).

The ELISA results show that seroconversions were observed at 10 dpi for the three goats (Figure 3A) and 12 to 14 dpi for cattle (Figure 3B). The ODs were all above 0.3 and were higher than 1 four weeks after BTV-4 inoculation. ELISA results obtained on 21 sheep and 55 cattle from the field (Table 1), which were BTV-4 SNT-positive because they were naturally infected, were all also ELISA-positive with OD values between 0.3 and 2 (data not shown). Based on these results and the results of the specificity study (Figure 2), we set the threshold for ELISA positivity at 0.25. OD < 0.25 is considered negative and OD ≥ 0.25 positive.

### 3.4. Detection of BTV-4-Specific Antibodies in Vaccinated Animals

Twelve out of the one hundred and eighty goats were vaccinated twice with a bivalent BTV-8 and four were found to be BTV-4 ELISA negative (Table 2). Eleven of these twelve sera were also BTV-4 SNT-negative and 1 positive. Thus, serotype-specific BTV-4 vaccine antibodies were detected in 93.3% (168/180) of these vaccinated goats. Surprisingly, 28 positive ELISA sera (with OD between 0.3 and 1.4) were previously found to be SNT-negative. Overall, 151 (83.8%) sera had concordant SNT and ELISA results.

Table 3 shows that ELISA was able to detect vaccine antibodies 21 days after primary BTV-4 vaccination in 12 of 15 vaccinated sheep.

Table 4 summarizes the ELISA results obtained on zoo species after vaccination. The results show that VP2 antibodies are well detected in these 15 different wild animal species (Table 3). Some of them seem to respond well to vaccination (chital deer, sable antelope, marshbuck, etc.) while for other species (Indian hog deer, Chinese muntjac), only a few individuals seroconvert following two injections of the bivalent BTV-4 vaccine. The BTV-4 SNT and ELISA results in this population were 91.7% in agreement (133/145).

### 3.5. ELISA Analytical Sensitivity

**Figure 4:**
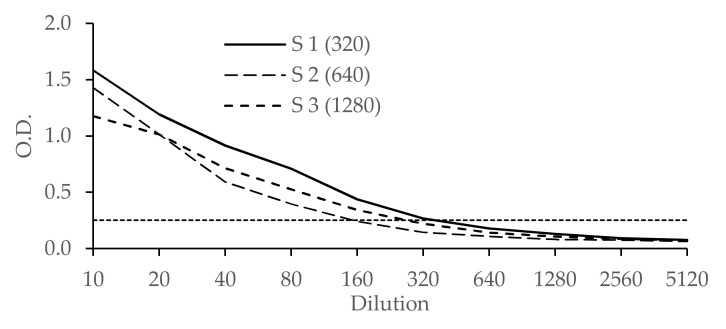
OD values of three BTV-4 ELISA-positive twofold serially diluted sera. In brackets the dilution up to which the serum was BTV-4 SNT-positive. The horizontal line represents the ELISA cut-off (OD = 0.25).

The analytical sensitivity of the ELISA is illustrated in Figure 4. Sensitivity was very satisfactory: all three sera (from infected animals) were ELISA-positive up to dilution 160.

### 3.6. ELISA Results with BTV Reference Sera

**Figure 5:**
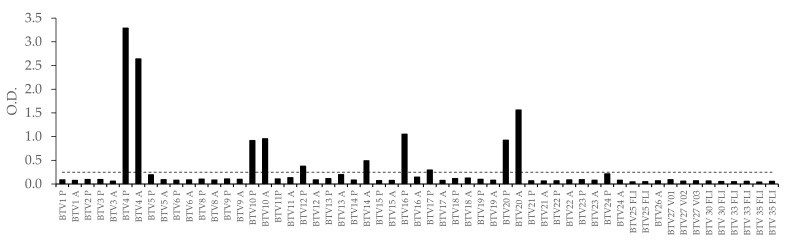
ELISA results (OD) in presence of BTV reference sera. A, P or FLI: serum from Australia (CSIRO), Pirbright or Friedrich-Loeffler-Institut, respectively. The horizontal line represents the ELISA cut-off (OD = 0.25).

In all, 57 reference sera were tested with the BTV-4 ELISA. Figure 5 illustrates the OD values obtained. The highest ODs were accessed with the two anti-BTV-4 reference sera, followed by the two BTV-10 and two BTV-20 sera. One of the two anti-BTV-16 sera from the Pirbright collection was ELISA-positive while the Australian anti-BTV-16 serum was negative. Similarly, one of the two anti-BTV-14 (Australian) sera was weakly positive (OD = 0.49), while the Pirbright collection serum was negative. Finally, weak positive signals were observed for one of the two anti-BTV-12, 17 and 24 sera. None of the sera from atypical strains (BTV-25–28, 30, 33 and 35) were found to be positive.

## 4. Discussion

Cloning of the VP2 coding sequence into the pVote plasmid was easily achieved by recombination using the Gateway^®^ cloning system. The green fluorescence of the recombinant T7 green VacV allowed easy visualization of virus propagation in BSR cells and facilitated the selection steps.

In order to perform a double-antigen sandwich ELISA, it was necessary to obtain tens of µg of highly purified recombinant VP2. Expression with the T7 green VacV enables us to obtain this quantity so that it could be used as an antigen to coat Maxisorb plates and also to carry out peroxidase labelling in such a way that, with 10 T75 flasks, it was possible to carry out several thousand ELISA points. Purification by immunoprecipitation with the FLAG tag also made it possible to obtain a highly purified recombinant protein (Figure 1, column 2) with very little or no protein from the BSR cultures, and this without using detergents. The purified recombinant VP2 conserves its conformation and therefore its antigenic properties.

The ELISA was developed using a commercial ELISA buffer (No 14, Innovative Diagnosis) diluted 1:4 in PBS, but a classical PBS tween milk buffer could also have been used with identical results. The use of either of these buffers results in very low background levels for all animal species tested. The use of a VP2-HRP also seems to provide high specificity, illustrated by the low background regardless of the animal species analyzed. Each BTV-4 VP2 has 58 lysines, which are targeted by the covalent binding of the HRP, and thus allow for significant signal amplification during assay revelation. This binding of HRP to lysines does not appear to interfere with antigen recognition by captured anti-VP2 antibodies.

The specificity of the ELISA evaluated in the presence of BTV antibody negative sera showed good results: all 306 sera had an OD < 0.25 (Figure 2A). The BTV-4 ELISA did not detect positive sera experimentally infected with the seven EHDV serotypes. The specificity of the test on this population was evaluated at 100% (confidence interval (CI): 98.7–100%).

Similarly, we particularly wanted to evaluate the specificity of the test in relation to BTV-8 infected sera (231 sera tested) in view of the French (and European) context. This serotype is predominantly present and in SNT some cross-reactions have been observed (false positives) [13,14]. In addition, sera infected or vaccinated with BTV-1, 2, 9 and 16 were also tested, as these serotypes are those circulating or that have circulated in Europe since the 2000s. Only one serum (out of the three hundred and nineteen tested) from a sheep experimentally infected with BTV-1 [27] was weakly positive (OD = 0.302) (Figure 2B). None of the five sera from animals experimentally infected with BTV-16, which were BTV-16 SNT-positive, reacted with the BTV-4 ELISA, nor did the Australian anti-BTV-16 reference serum (Figure 4). Only the Pirbright BTV-16 serum was detected positive (OD = 1.05), but based on the results of the five BTV-16 sera from sheep challenged with the GRE2008/11 strain isolated in 2008 from blood of an infected Greek sheep [18], we believe that our BTV-4 ELISA does not react with the BTV-16 VP2 antibodies generated with this European strain. The BTV-16 Pirbright reference serum tested in this study has been in our laboratory for several decades. The strain used for its manufacture cannot be one of the BTV16 strains circulating in Europe after the 2000s, but most certainly of African or Asian origin. Thus, out of these 625 ‘non-BTV-4’ sera tested, the specificity is 99.84% (CI: 99.1–99.7%). Concerning the BTV-3 present in the Mediterranean basin [28,29], only two reference sera were available and were found to be ELISA BTV-4-negative. Therefore, this test seems to be suitable for use in the European and Mediterranean area, not interacting with serotypes 1, 2, 3, 8, 9 and 16, as well as with atypical BTV serotypes detected in Europe (BTV-25, 27).

Figure 3 shows that anti-VP2 antibodies are detected 10 days (goats) or 12–14 days (cattle) after inoculation, i.e., 4 days after the detection of anti-VP7 antibodies in cELISA [19], indicating that the VP7 seroconversion occurred before the appearance and the detection of NA.

In this study, the NA titer of a serum was defined as the highest dilution allowing neutralization of the 100 TCID50. We report an SNT-positive result when a serum has an SNT BTV-4 titer ≥ 1 (log10 of the dilution 10). With reference to this SNT threshold, three goats were BTV-4 SNT-positive at D8, D10 and D12 and two cattle were positive at D12 and the third at D17. A comparison between the SNT and ELISA results (Figure 3A,B) suggests that the BTV-4 ELISA can detect the first anti-VP2 antibodies at least as early as SNT.

After ELISA seroconversion (Figure 3), all the sera tested were found to be ELISA-positive until D63 post-inoculation. In the same way, the 76 sera from field animals found to be BTV-4 ELISA-positive were also BTV-4 SNT-positive (Table 1). However, results from the three diluted sera (Figure 4), from infected ruminants suggested that the sensitivity of the ELISA was lower than the SNT: they were found to be BTV-4 ELISA-positive up to dilution 160–320, whereas they were detected positive by BTV-4 SNT until titers ranging from 320 to 1280. This better sensitivity of the SNT may be explained by the fact that SNT uses larger volumes of serum than ELISA, as observed for the double-antigen microsphere immunoassay for simultaneous group and serotype detection of BTV antibodies [15]. In view of all these BTV-4 SNT and ELISA results, the sensitivity of the ELISA, from infected animal sera, appears to be highly satisfactory. However, too few BTV-4-positive samples (from the field or experiments) could be tested in this study to finely evaluate and quantify the sensitivity of the test.

The BTV-4 ELISA test is also effective in detecting vaccine antibodies. Results from the 180 goats vaccinated with a bivalent inactivated BTV-8 and 4 vaccines show that the ELISA detects 93.3% of the vaccinated animals (168/180) (Table 2). Surprisingly, 28 of the 168 BTV-4 ELISA-positive sera were previously found to be BTV-4 SNT-negative (and pan-BTV-ELISA-positive); the other 140 were SNT-positive. These results suggest that BTV-4 ELISA is more effective than SNT in detecting vaccine antibodies but are in contrast to the observations obtained from sera of naturally or experimentally infected animals, where SNT appeared to be at least as sensitive as ELISA. This difference could be due to the fact that the immune response of an infected host is not the same as that of an animal vaccinated with an inactivated vaccine and/or to variations in the SNT method with respect to the different batches of BTV-4 strains used over time (here several years) to perform the different SNT series.

In 15 sheep vaccinated with an inactivated vaccine (Table 3), 12 were found to be positive 21 days after the first BTV-4 injection, and all those tested 7 or 21 days after the second injection were positive. These results confirm that ELISA is an effective tool for detecting vaccine antibodies in sheep. Non-domestic animals, here from zoos, vaccinated for export, were found to be ELISA-positive in varying proportions depending on the genus/species tested (Table 4). Animals belonging to the genus blue wildebeest, camel or dik dik appear not to have anti-VP2 antibodies after vaccination. In the *Axis* species, chital deer (*n* = 33) all seroconvert after vaccination, whereas only three Indian hog deer out of the eight vaccinated were BTV-4 ELISA-positive. However, too few zoo animals were tested in these different genera to be certain of their susceptibility (or non-susceptibility) to inactivated bluetongue vaccines. Apart from the fact that these zoo animals were vaccinated with a bivalent BTV-4 and 8 vaccine, it is not known whether they were previously infected with wild BTV-8, which has been widespread in France since 2015, and thus the 7 SNT-positive (Table 4) and ELISA-negative sera could be due to previous infection with BTV-8 and thus to non-specific BTV-4 SNT reactions observed in the presence of BTV-8 infected animals’ sera [13,14]. Overall, the ELISA results from the 145 vaccinated zoo animals showed 93.8% agreement with the SNT results. As observed for the vaccinated goats, five SNT-positive sera were found to be ELISA-negative.

The results obtained from vaccinated animals (from the field or zoos) could not be used for the sensitivity evaluation. In particular, we did not know whether the different species of wild animals in zoos are susceptible or not to BTV (or to inactivated vaccines), and we did not know the BTV status of the 180 goats prior to vaccination. Concerning the 28 vaccinated goats and the 5 vaccinated zoo animals that were BTV-4 ELISA-negative but SNT-positive, these results are in contrast to observations obtained from sera of naturally or experimentally infected animals, where SNT appeared to be as sensitive (or even more so) than ELISA. However, the overall results obtained on domestic ruminants show that this test detects a high proportion of those vaccinated with inactivated BTV-4 vaccines. Furthermore, based on the results obtained from the experiments performed to evaluate the specificity of the ELISA, we prefer to take into account the ELISA results for samples with positive SNT and negative ELISA results.

The BTV-4 ELISA results obtained from the reference sera confirm the serotype relationships described by Erasmus in 1990 [30]. Figure 5 shows that the two reference sera for serotypes 10 and 20 cross-react the most with the BTV-4 ELISA. One of the two anti-BTV-12, 17 and 24 sera, genetically closely related to serotype 4, have ELISA results close to the threshold of positivity, indicating weak recognition of anti-BTV-12, 17 and 24 antibodies with the recombinant BTV-4 VP2. A serum identified as anti-BTV-14 from Australia was also found to be positive in the BTV-4 ELISA, although the potential cause of this positive result cannot be fully explained (as observed with the anti-BTV-16 from Pirbright). In order to increase the specificity of the test with respect to strains genetically close to serotype 4, it is sufficient to dilute the sera (from infected animals) to 1:25 or 1:50; the analytical sensitivity of the ELISA (Figure 4) then allows the specific characterization of BTV-4 infected animals, to the detriment of detecting vaccinated animals.

## 5. Conclusions

The overall results show that the double antigen ELISA developed can be used to screen for BTV-4-specific antibodies from susceptible animals reared in Europe and the Mediterranean basin. The specificity of the test is excellent for the specific detection of BTV-4 vaccinated or infected animals; however, the test can yield positive results with sera from animals infected with serotypes 10, 12, 17 and 20, which have never been detected in Europe and the Mediterranean basin [4]. The sensitivity of the developed ELISA is also satisfactory: the test detected the presence of anti-VP2 antibodies 10 to 14 days after BTV-4 inoculation and 3 weeks after a first vaccine injection. This ELISA is more reliable for the determination of the presence of antibodies specific to serotype 4 than the SNT method in the current context, where serotype 8 induces cross-reactions in BTV-4 SNT, and therefore false positives. Our objectives are to develop and evaluate a BTV-8-specific double antigen ELISA and in the longer term to replace the diagnosis of BTV-4-positive sera by these serotype-specific ELISAs.

## Figures and Tables

**Table 1:** Origin, status of the animals and year of sampling/article references. IRM: Internal Reference Material for ELISA.

Origin	Animals	Status	Serum Number	Country	Year of Sampling/Ref	Total
field	cattle	Naïve	96	France	1998–2000	319
	goats		110		2006	
	sheep		64		2008–2015	
experimental assay	sheep	Naïve	26	France	[18]	
Field	deer	Naïve	5	Guyana	2020	
experimental assay	cattle, goats	Naïve	11	Germany	[19]	
	cattle	EHD	7	France	[20]	
experimental assay	sheep	BTV-2	8	France	2004	306
	sheep	BTV-1	41		[21,22]	
experimental assay	sheep	BTV-9	21	France	[18]	
	sheep	BTV-16	5			
field	cattle	BTV-8	202		2015	
	sheep		10			
experimental assay	cattle, goats, rabbit	BTV-8	16	Germany	-	
	MRI		3	France	-	
experimental assay	cattle	BTV-4	27	Germany	[19]	141
	goats		35			
field	cattle	BTV-4	55 *	France	2018–2021	
	sheep		21 *		2017	
experimental assay	sheep	BTV-4	3	Germany	-	
field	goats	vaccinated BTV-4	180	France	2019	363
experimental assay	sheep		38		[18]	
zoo	see Table 3		145		2018–2021	
reference sera	23	BTV-1-6, 8-24	23	UK (Pirbright)	-	57
	23	BTV-1, 2-6, 8-24, 26	23	Australia	-	
	3	BTV-27	3	France	-	
	8	BTV-25, 30, 33, 35	8	Germany	-	

*: BTV-4 SNT positive sera.

**Table 2:** BTV-4 ELISA and SNT-4 results from 180 vaccinated goats.

		SNT Results		
		Positive	Negative	Total
BTV-4 ELISA results	positive	140	28	168
	negative	1	11	12
	total	141	39	180

**Table 3:** ELISA results from sheep sera vaccinated with inactivated vaccines.

	Days after BTV-4 Vaccination (Number of BTV-4 ELISA Positive/Total Tested)			
	5	21	28	42
Vac 2 and 4 (2x)	(0/4)a	(5/5)b	(5/5)b	(4/4)b
Vac 9, 2 and 4 *	(0/8)	(7/10)	-	-

Vac 2 and 4: vaccinated twice (at D0 and D21) with a bivalent BTV-2 and 4 vaccine. Vac 9, 2 and 4: vaccinated with a monovalent BTV-9 vaccine (D-21) and then with a bivalent vaccine BTV-2 and 4 (at D0). a: all sera were VP7 ELISA negative, b: all sera were VP7 ELISA positive. *: no interpretation possible between VP7 seroconversion and BTV-4 vaccination (animals previously vaccinated with inactivated BTV-9).

**Table 4:** BTV-4 ELISA results from 15 zoo genera.

			ELISA BTV-4 Results		
Zoo Species		Number of Animals	Negative	Positive	% Positive
Antilope cervicapra	Blackbuck	39	6	33	84.6
Axis axis	Chital deer	33	0	33	100
Axis porcinus	Indian hog deer	8	5	3	37.5
Budocas taxicolor	Takin Cattle	5	1	4	80
Camelus dromedarius	Arabian camel	2	2	0	0
Connochaetes taurinus	Blue wildebeest	2	2	0	0
Hippotragus niger	Sable antelope	10	1	9	90
Madoqua kirkii	Kirk’s dik-dik	2	2	0	0
Muntiacus reevesi	Chinese muntja	12	5	7	58.3
Oryx beisa	East African oryx	3	1	2	66.7
Tragelaphus angasii	Nyala	3	0	3	100
Tragelaphusus eurycerus	Bongo	7	2	5	71.4
Tragelaphus imberbis	Lesser kudu	3	0	3	100
Tragelaphus spekii	Marshbuck	14	3	11	78.6
Tragulus javanicus	Lesser mouse-deer	2	0	2	100
total		145	30 *	115 **	

*: 7/30 were BTV-4 SNT-positive; **: 5/115 were BTV-4 SNT-negative.

## Data Availability

The data that support the findings of this study are available from the corresponding author upon reasonable request.
